# Development of novel target modules for retargeting of UniCAR T cells to GD2 positive tumor cells

**DOI:** 10.18632/oncotarget.21017

**Published:** 2017-09-18

**Authors:** Nicola Mitwasi, Anja Feldmann, Ralf Bergmann, Nicole Berndt, Claudia Arndt, Stefanie Koristka, Alexandra Kegler, Justyna Jureczek, Anja Hoffmann, Armin Ehninger, Marc Cartellieri, Susann Albert, Claudia Rossig, Gerhard Ehninger, Jens Pietzsch, Jörg Steinbach, Michael Bachmann

**Affiliations:** ^1^ University Cancer Center (UCC) ‘Carl Gustav Carus’ TU Dresden, Tumor Immunology, Dresden, Germany; ^2^ Helmholtz-Zentrum Dresden-Rossendorf (HZDR), Institute of Radiopharmaceutical Cancer Research, Dresden, Germany; ^3^ German Cancer Consortium (DKTK), partner site Dresden, and German Cancer Research Center (DKFZ), Heidelberg, Germany; ^4^ GEMoaB Monoclonals GmbH, Dresden, Germany; ^5^ Cellex Patient Treatment GmbH, Dresden, Germany; ^6^ Department of Pediatric Hematology and Oncology, University Children’s Hospital Münster, Münster, Germany; ^7^ Medical Clinic and Policlinic I, University Hospital ‘Carl Gustav Carus’, TU Dresden, Dresden, Germany; ^8^ National Center for Tumor Diseases (NCT), Dresden, ‘Carl Gustav Carus’ TU Dresden, Dresden, Germany; ^9^ Department of Chemistry and Food Chemistry, School of Science, ‘Carl Gustav Carus’ TU Dresden, Germany

**Keywords:** immunotherapy, CAR T cells

## Abstract

As the expression of a tumor associated antigen (TAA) is commonly not restricted to tumor cells, adoptively transferred T cells modified to express a conventional chimeric antigen receptor (CAR) might not only destroy the tumor cells but also attack target-positive healthy tissues. Furthermore, CAR T cells in patients with large tumor bulks will unpredictably proliferate and put the patients at high risk of adverse side effects including cytokine storms and tumor lysis syndrome. To overcome these problems, we previously established a modular CAR technology termed UniCAR: UniCAR T cells can repeatedly be turned on and off via dosing of a target module (TM). TMs are bispecific molecules which cross-link UniCAR T cells with target cells. After elimination of the respective TM, UniCAR T cells automatically turn off. Here we describe novel TMs against the disialoganglioside GD2 which is overexpressed in neuroectodermal but also many other tumors. In the presence of GD2-specific TMs, we see a highly efficient target-specific and -dependent activation of UniCAR T cells, secretion of pro-inflammatory cytokines, and tumor cell lysis both *in vitro* and experimental mice. According to PET-imaging, anti-GD2 TM enrich at the tumor site and are rapidly eliminated thus fulfilling all prerequisites of a UniCAR TM.

## INTRODUCTION

Neuroblastoma is the most frequent extracranial tumor occurring in young children. Half of the patients have metastatic disease at the time of diagnosis. Prognosis for patients with disseminated disease is poor underlining the need for innovative therapies [1, 2]. The disialoganglioside GD2 is highly expressed in neuroblastoma and also several other pediatric as well as adult cancers, for example in melanoma, osteosarcoma, uterine leiomyosarcoma, small cell lung cancer, Ewing’s sarcoma, and retinoblastoma. GD2 is also expressed on healthy tissues especially during fetal development. In healthy post-natal tissues GD2 expression is low and limited on skin melanocytes, osteoprogenitors, peripheral nerves and the brain [3–11]. As GD2 expression is high on tumor cells but low on normal tissues, GD2 has become an interesting target for immunotherapeutic approaches including monoclonal antibodies (mAbs) and more recently chimeric antigen receptors (CARs). [12–17].

CARs are recombinant synthetic receptors. Like physiological receptors, CARs consist of three domains: (i) an extracellular binding moiety, (ii) a transmembrane domain and (iii) an intracellular domain containing signaling motif(s). The extracellular antigen binding moiety is commonly a single-chain fragment variable (scFv) recombinantly constructed by fusion of the variable heavy and light chain sequences from a mAb. The transmembrane domain is most frequently derived from the CD28 or CD8 receptor [18–22]. The intracellular signaling domains (ITAM motifs) are taken from activating immune receptors. The idea of chimeric antibody derived T cell receptors was first published in 1989 [23]. Since then, a variety of CAR designs were described including first, second and third generation CARs which differ with respect to their intracellular signaling domain(s) [18–22]. The clinical applications of CD19-specific CAR T cells underline both their impressive efficacy but also their high potential risk of severe side effects [24–27]. As CD19 is also expressed on healthy B cells, B cell lymphoma patients treated with CD19 CAR T cells develop ongoing B cell aplasia. While the lack of B cells is manageable by intravenous immunoglobulin administration, in the case of GD2, as with other TAAs, such on-target, off-tumor effects may not be acceptable or even become life-threatening [28, 29]. The most relevant toxicity of GD2 antibody targeting is a generalized pain syndrome attributed to GD2 antigen expression on peripheral nerves [13]. CAR T cells, other than mAbs, are able to penetrate the blood brain barrier and exert activity against their target cells in the CNS [27, 30]. While CAR T cells in the first clinical studies have not shown any neurotoxic side effects [14], advanced and more effective CAR T cell designs may lead to damaging side effects by cotargeting GD2^low^-expressing cells in the central or peripheral nervous system.

**Figure 1:**
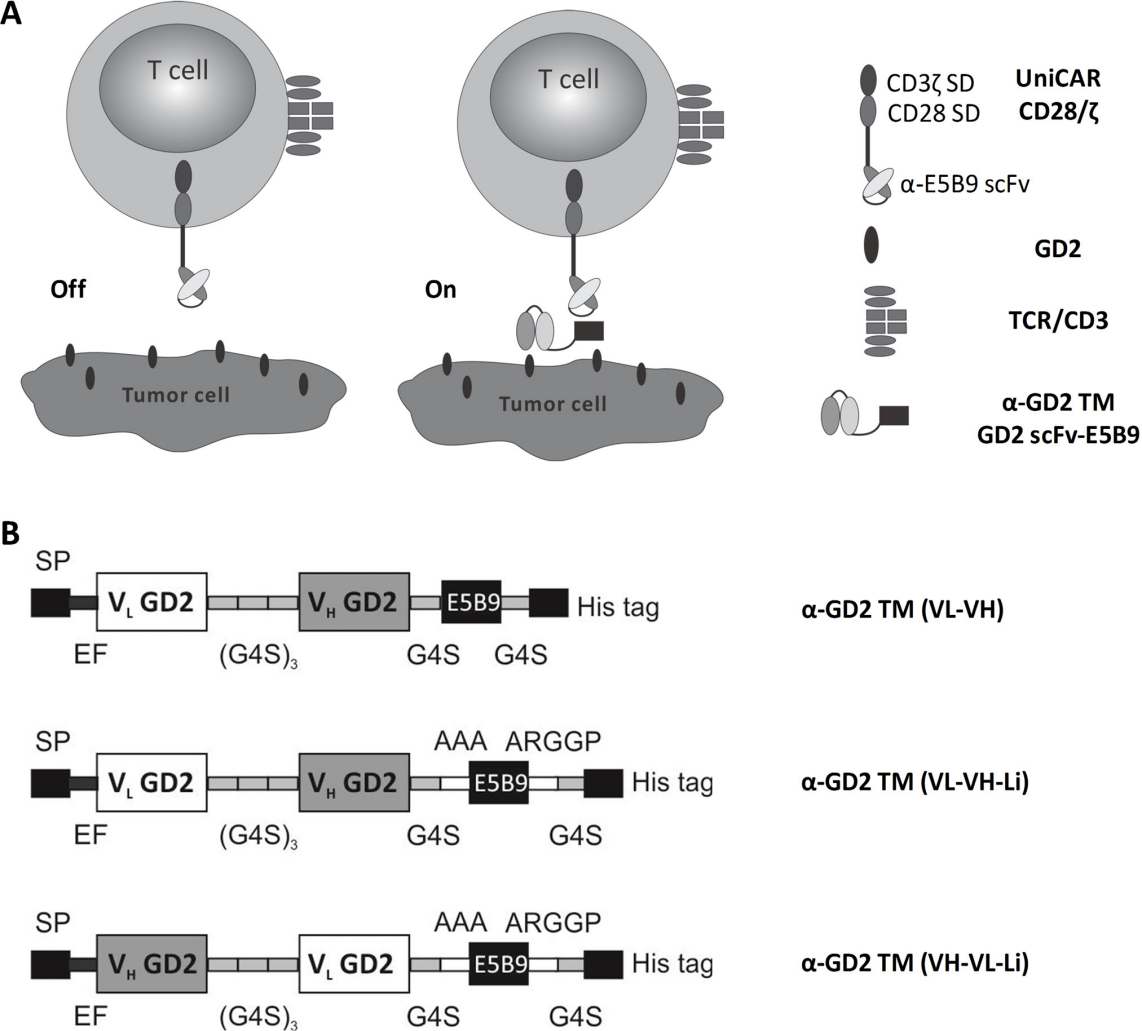
Construction of novel α-GD2 TMs for redirecting UniCAR T cells to GD2 positive tumor cells (**A**) Schematic summary of the UniCAR principle. In the absence of a TM UniCAR T cells are inactive (Off). In the presence of a TM UniCAR T cells can interact with target cells (On). For this purpose, TMs are bispecific molecules. On the one hand, TMs can bind to a cell surface target antigen (here GD2). On the other hand, they can form a complex with the extracellular binding domain of UniCARs via a peptide epitope (E5B9, UniCAR epitope). (**B**) Schematic view of the structure of the three novel α-GD2 TMs: In the first (α-GD2 TM VL-VH) and the second (α-GD2 TM VL-VH-Li) construct the VH and VL sequences were arranged in VL-VH orientation, in the third (α-GD2 TM VH-VL-Li) construct in VH-VL orientation. The UniCAR epitope (E5B9) was fused to the C-terminus of the scFv either directly (α-GD2 TM VL-VH) or flanked by two spacer peptides (N-terminal spacer: AAA; C-terminal spacer: ARGGP) (α-GD2 TM VL-VH-Li, α-GD2 TM VH-VL-Li).

To prevent unmanageable toxicities by the *in vivo* persistence of CAR T cells, in 2014 we introduced a modular CAR platform technology which we termed universal CAR (UniCAR) [31]. A schematic view of the UniCAR principle is shown in Figure 1A. The UniCAR system originated from our previously described modular BiTE (Bispecific T cell engager) format [32–34]. In contrast to conventional CAR T cells, UniCAR T cells are not directed to a cell surface epitope but recognize a unique peptide epitope. Therefore, UniCAR T cells per se are inert but can repeatedly be turned on and off via dosing of a target module (TM). TMs in general are bispecific molecules which cross-link UniCAR T cells with target cells: TMs are fusion molecules consisting of the peptide epitope recognized by UniCARs and a binding domain directed against the TAA. Due to the modular character UniCAR T cells can reversibly be armed with one or even multiple TMs [31, 35–37]. Side by side comparison shows that the killing capability of UniCAR T cells armed with TMs does not differ from conventional CAR T cells [36]. UniCAR/TM complexes can reversibly and rapidly associate and dissociate in dependence on the concentration of the TM. Unbound TMs are rapidly eliminated from peripheral blood [36, 37]. Therefore, we expect that UniCAR T cells in clinical use will automatically be switched off when the respective TM is eliminated from a patient, thus providing a self-limiting safety switch. For this reason, the UniCAR system is an attractive platform for targeting of TAAs which are highly expressed on tumors but to some extent also on critical healthy tissues such as GD2.

Here we show proof of concept for both *in vitro* and *in vivo* retargeting of GD2 positive tumor cells with UniCAR T cells armed with anti-GD2 TMs.

## RESULTS

### Construction of TMs directed against GD2

So far all TMs described in our previous studies were directed against protein targets including CD33, CD123 [35] in leukemias and PSCA, PSMA [36] and EGFR [37] in solid tumors. All these TMs were cloned in either a single chain fragment variable (scFv) [35, 36] or nanobody [37] format. The novel TMs against the disialoganglioside GD2 were constructed starting from the sequence of the variable heavy and light chains of a previously described conventional anti-GD2 CAR [13]. As schematically summarized in Figure 1B, three anti-GD2 TMs were constructed by fusing the UniCAR epitope to the respective anti-GD2 scFv: In one TM the variable chains of the scFv were rearranged in the orientation VL-VH (Figure 1B, α-GD2 TM (VL-VH)). In the second TM the variable domains were organized in the same way. To increase the distance of the UniCAR epitope (see MATERIALS AND METHODS) to both the scFv portion and the C-terminal oligo-his tag, two spacer peptide sequences were inserted: One N- (AAA) the other one C-terminally (ARGGP) of the UniCAR epitope (Figure 1B, α-GD2 TM (VL-VH-Li)). In the third TM the UniCAR peptide epitope was flanked in the same way, and the variable domains in the scFv were arranged in VH-VL orientation (Figure 1B, α-GD2 TM (VH-VL-Li)).

### Expression and isolation of anti-GD2 TMs

For expression the reading frames encoding the TMs were transduced into CHO cells, and permanent cell lines expressing the respective TM were established as described previously [38, see also MATERIALS AND METHODS]. TMs were purified from cell culture supernatants of the eukaryotic cells using Nickel affinity chromatography [36, see also MATERIALS AND METHODS].

**Figure 2:**
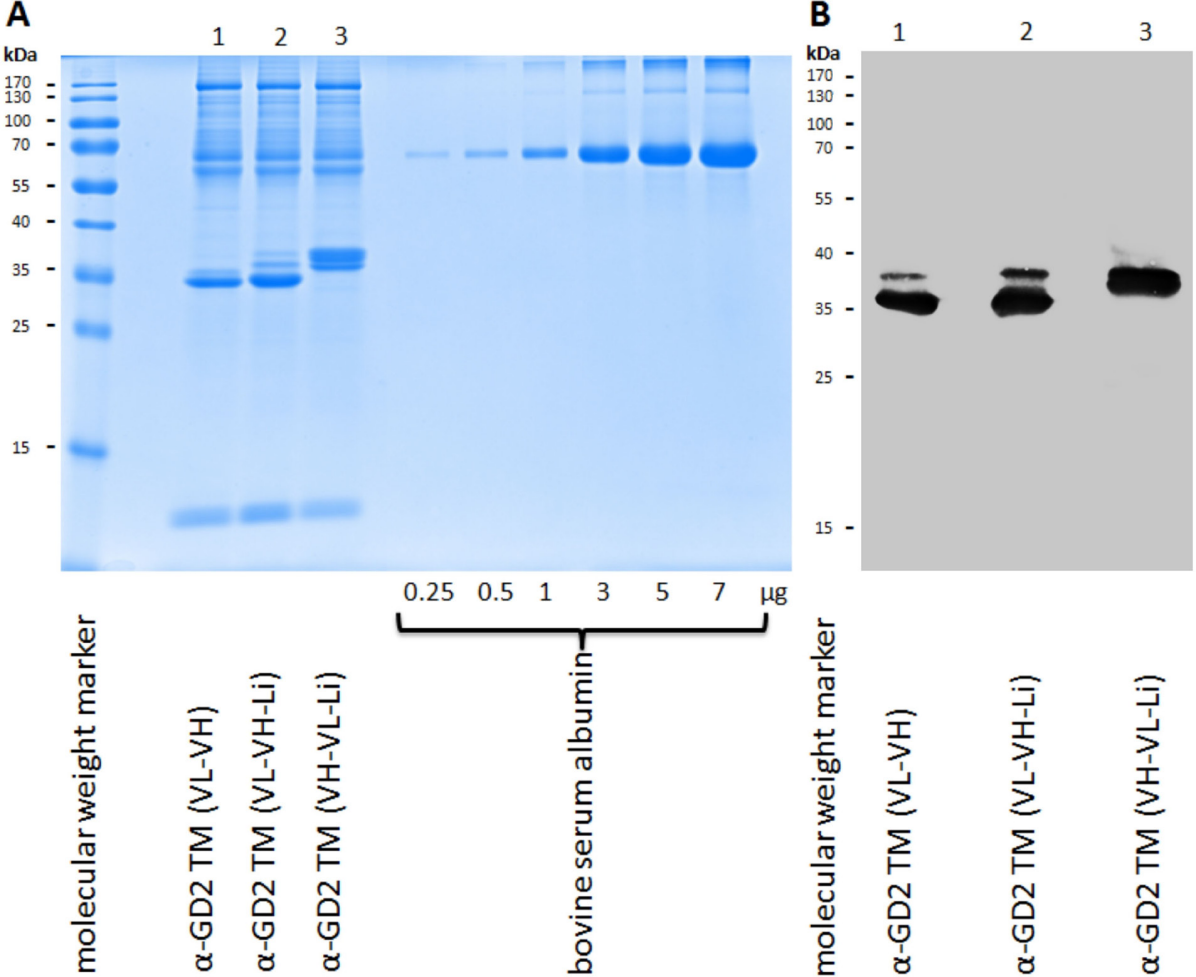
Expression and analysis of affinity-purified α-GD2 TMs All three α-GD2 TMs were expressed using CHO cells. The CHO cells were transduced with the open reading frame encoding one of the three α-GD2 TMs (lane 1, α-GD2 TM VL-VH; lane 2, α-GD2 TM VL-VH-Li; lane 3, α-GD2 TM VH-VL-Li). The eluted TMs were separated via SDS-PAGE and subsequently stained with Coomassie Brilliant Blue G250 (**A**), or transferred onto a nitrocellulose membrane for detection with the mAb directed against the UniCAR epitope (**B**).

**Figure 3:**
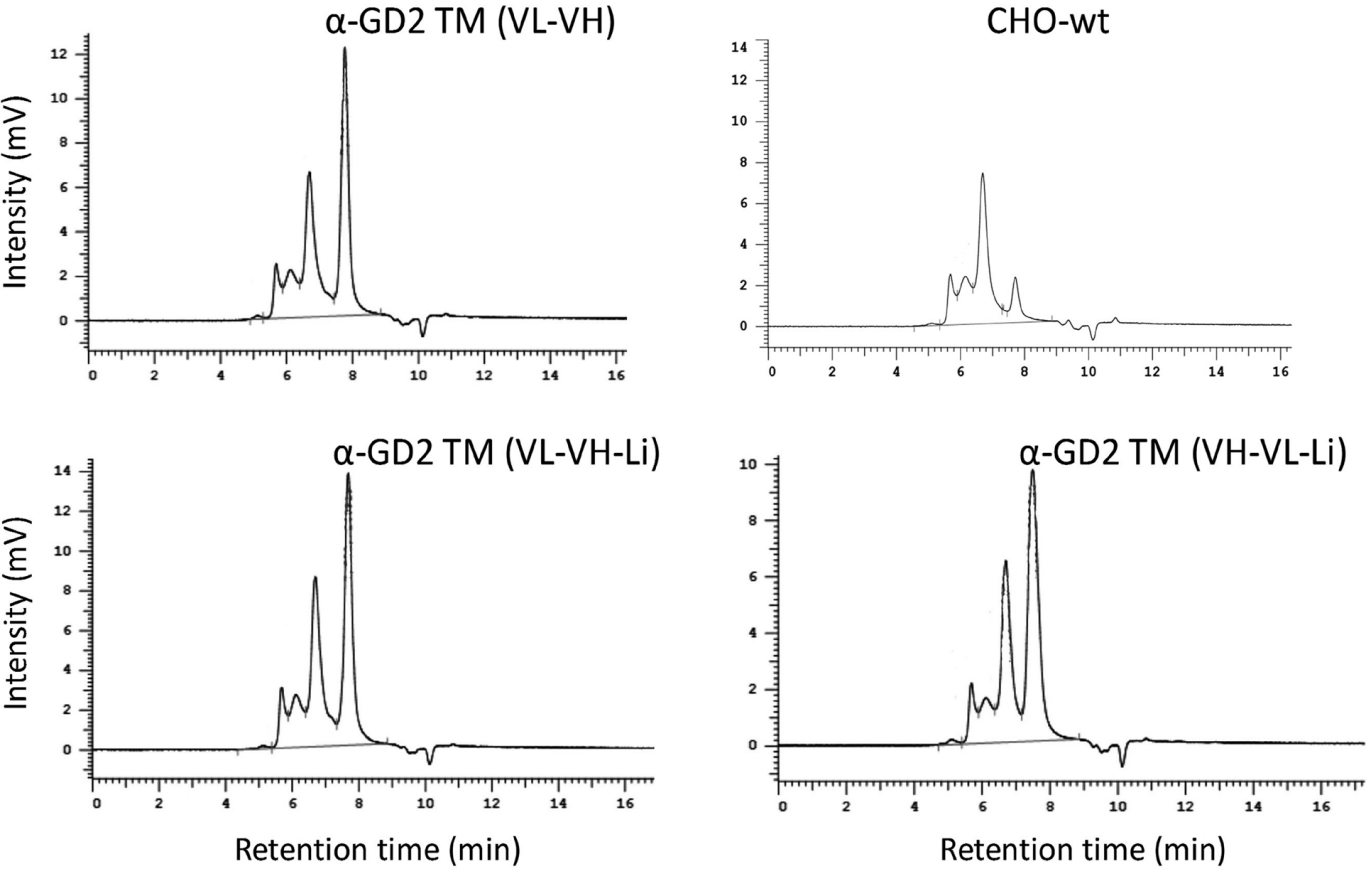
Analysis of affinity-purified α-GD2 TMs by HPLC size exclusion chromatography The three α-GD2 TMs were analyzed by SE-HPLC as described previously [37, see also MATERIALS AND METHODS].

In a first step the isolated TMs were biochemically analyzed by SDS-PAGE (Figure 2A, lanes 1 to 3) and immunoblotting (Figure 2B, lanes 1 to 3). As shown in Figure 2A, the three TMs are well expressed and, after heat denaturation, the UniCAR epitope is accessible for the mAb directed against the UniCAR epitope (UniCAR tag) (Figure 2B). Although the theoretical molecular weights of the three constructs do not differ substantially, for an unknown reason the α-GD2 TM (VH-VL-Li) (Figure 2A, lane 3) shows an aberrant mobility. As already seen by SDS-PAGE, all the isolated TMs are contaminated with higher molecular weight protein species (HMWs). After SDS-PAGE/immunoblotting none of these HMWs reacted with the mAb directed against the UniCAR epitope (Figure 2B). Therefore, these HMWs should not be related to the α-GD2 TMs. Furthermore, they should not represent aggregates formed by dimerization or oligomerization of the scFv (for a more detailed analysis see below). This interpretation is in line with data obtained by HPLC chromatography (Figure 3). When cell culture supernatant of wildtype CHO cells is purified in the same way as the supernatant of cell lines expressing a TM, and the eluted fraction is analyzed by HPLC, a similar HMW pattern is obtained. Consequently, these non-immunoreactive HMWs are most likely contaminations deriving from CHO cells and/or fetal calf serum but not derived from α-GD2 TMs.

### Binding of the anti-GD2 TMs to GD2 positive tumor cells

**Figure 4:**
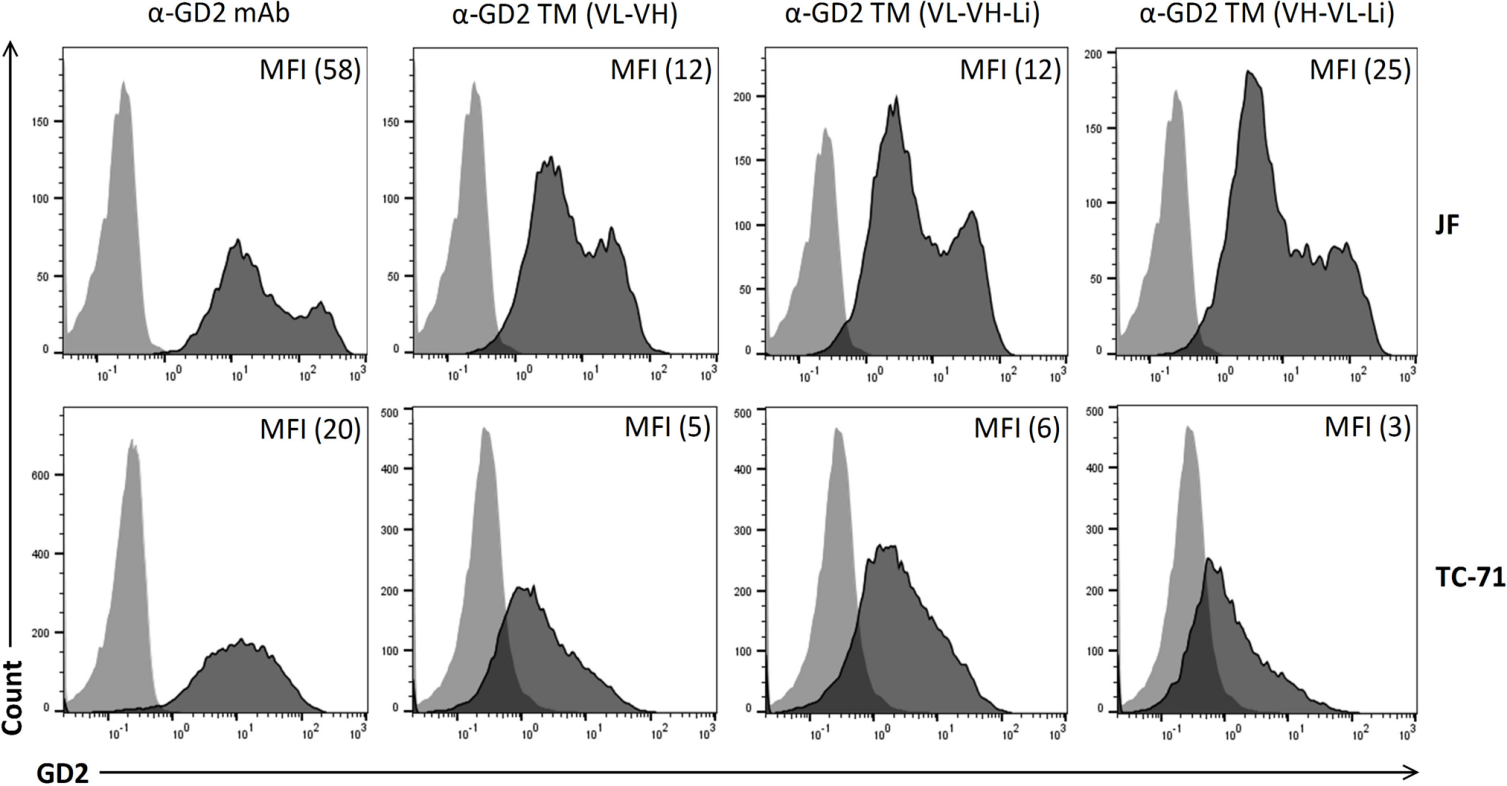
Binding of the α-GD2 TMs to GD2-expressing tumor cells (Upper panel, JF cells; Lower panel, TC-71 cells). Expression of GD2 was estimated by FACS analysis: Cells were stained with either a commercially available α-GD2 mAb and detected with Alexa Flour 647-conjugated anti-mouse-IgG mAb (dark graphs) or with the negative control Ab (light graphs). To verify the binding of the three α-GD2 TMs (α-GD2 TM VL-VH, α-GD2 TM VL-VH-Li, α-GD2 TM VH-VL-Li), the cells were stained with the respective TM. Their specific binding was detected via the mAb directed to the UniCAR epitope and Alexa Flour 647-conjugated goat anti-mouse IgG. (MFI) mean fluorescence intensity of stained cells.

**Figure 5:**
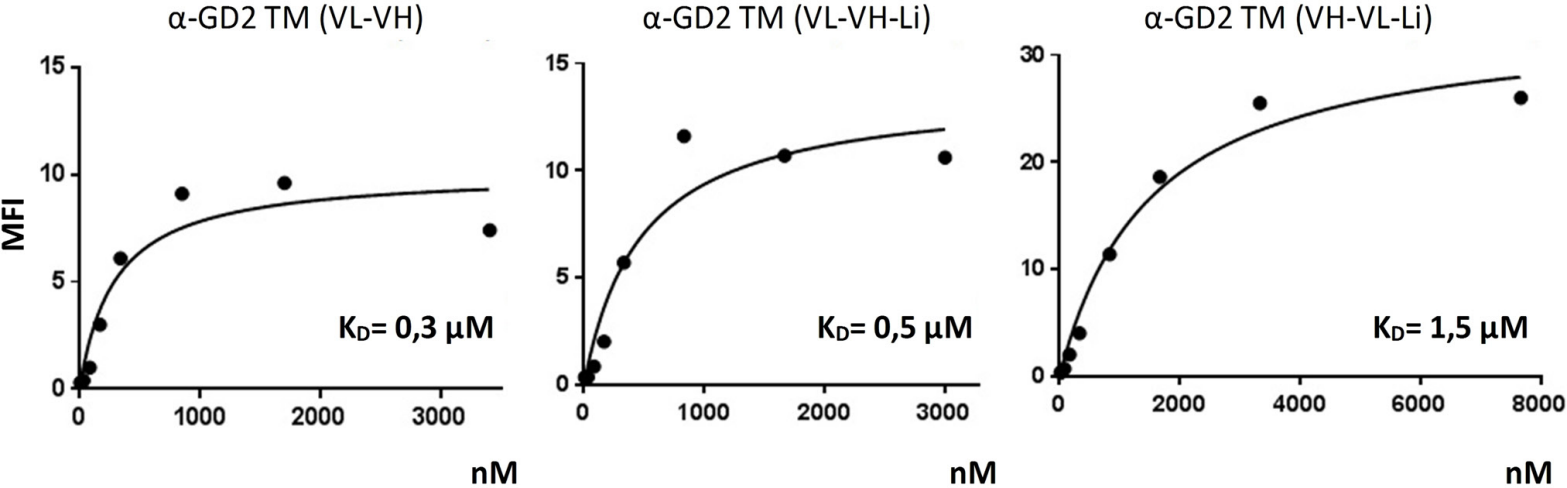
Estimation of *K*_D_ values For estimation of the *K*_D_ values of the novel α-GD2 TMs, increasing amounts of the respective α-GD2 TMs were used for staining of GD2 positive JF cells. Binding was detected via the mAb directed against the UniCAR epitope and Alexa Flour 647-conjugated goat anti-mouse IgG. The respective *K*_D_ value was calculated from the resulting binding curve [37]. MFI, mean fluorescence intensity of stained cells.

In the GD2 positive neuroblastoma cell line JF, GD2 surface expression was detected by FACS analysis using a commercial anti-GD2 mAb (Figure 4, upper panel, anti-GD2 mAb). Comparable data were obtained for the GD2 positive Ewing’s sarcoma cell line TC-71 (Figure 4, lower panel). According to the MFI values the expression level of GD2 is higher in JF cells compared to TC-71 cells. Moreover, JF cells contain GD2 high and low expressing populations. All three anti-GD2 TMs show comparable binding to JF- or TC-71 cells, respectively (Figure 4). In order to estimate their binding affinity, *K*_D_ values were estimated as described previously [36]. We determined a *K*_D_ value of 0.3 µM for the α-GD2 TM (VL-VH), 0.5 µM for the α-GD2 TM (VL-VH-Li), and 1.5 µM for the α-GD2 TM (VH-VL-Li) (Figure 5). According to these data (i) all the constructed anti-GD2 scFv domains are able to bind their target antigen and (ii) the UniCAR epitope is accessible in all the anti-GD2 TMs.

### Killing of GD2 positive tumor cells by retargeted UniCAR T cells occurs in a TM-Dependent and target-specific manner

**Figure 6:**
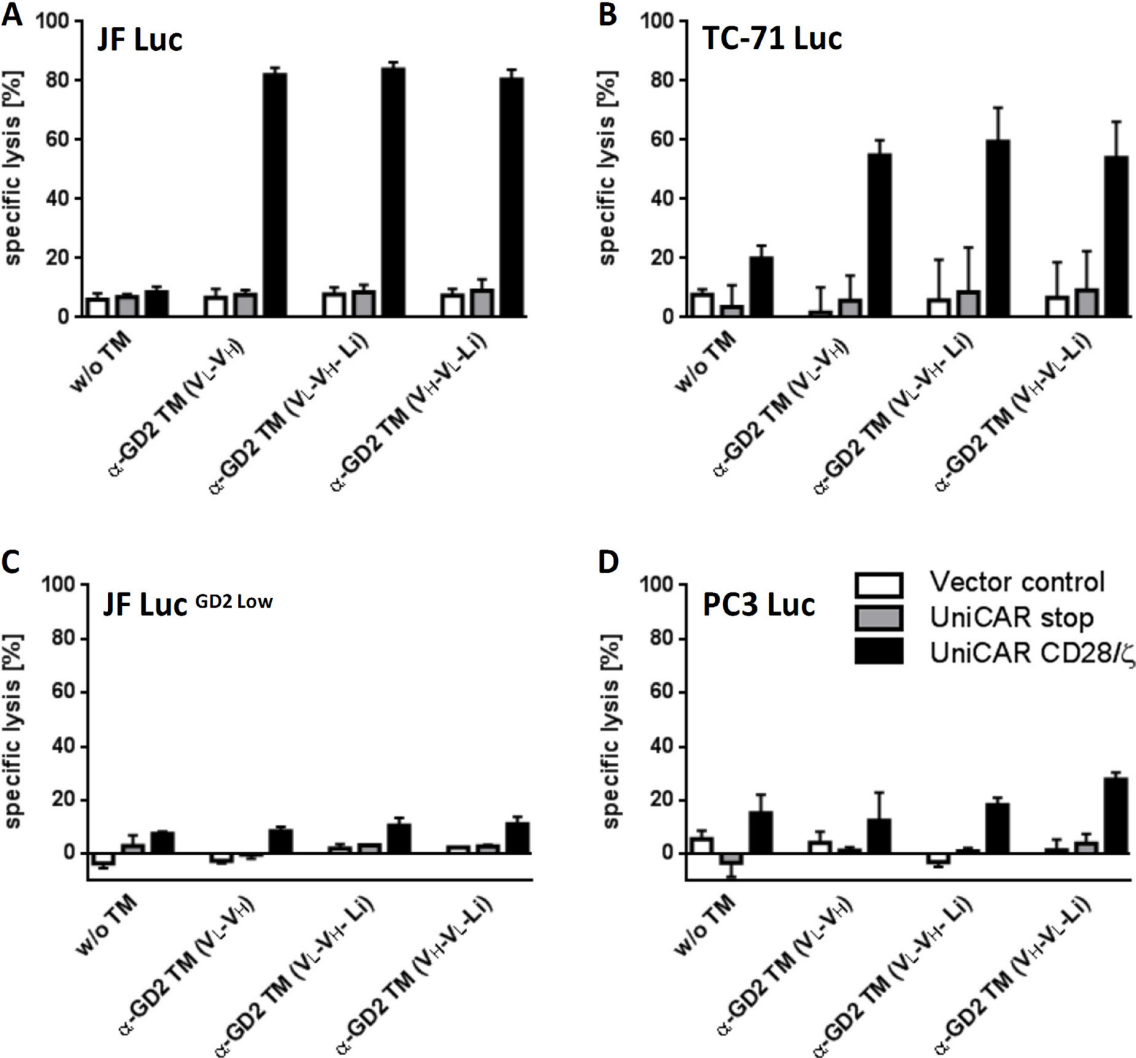
Retargeting of UniCAR T cells against GD2-positive tumor cells (**A–D**) For lysis assays four cell lines including (A) JF Luc cells, (B) TC-71 Luc cells, (C) JF Luc^GD2 low^, and (D) PC3-Luc cells expressing firefly luciferase were established. All cell lines were incubated with human T cells which were transduced with UniCARs containing either a dual CD28/CD3ζ signaling domain (UniCAR CD28/ζ) or lacking any signaling domain (UniCAR stop) or expressing only EGFP marker protein (Vector control). The respective UniCAR T cells were armed with 50 nM of one of the three α-GD2 TMs (α-GD2 TM VL-VH, α-GD2 TM VL-VH-Li, α-GD2 TM VH-VL-Li). The killing of the target cells was estimated using a Luciferase based bioluminescence assay. Mean and s.d. from experiments with three individual T cell donors are shown.

For functional analysis, human T cells from healthy donors were transduced with lentiviral vectors encoding the UniCAR sequence containing a dual CD28/CD3ζ signaling domain (Figure 6, UniCAR CD28/ζ). As negative control, T cells were transduced with lentiviral vectors encoding the UniCAR sequence lacking the signaling domain (Figure 6, UniCAR Stop). As additional negative controls served T cells transduced with a vector encoding EGFP marker protein (Figure 6, Vector control). For first lysis studies chromium release assays were performed (Data not shown): In the presence of the anti-GD2 TMs, retargeting of UniCAR T cells resulted in a specific lysis of both target cell lines (JF and TC-71). However, the labeling of both cell lines with chromium was critical as a chromium leakage occurred also in the absence of T cells. For a more reliable killing analysis we therefore adapted a Luciferase based killing assay. For this purpose, both GD2 positive cell lines (JF and TC-71) were transduced with the gene encoding firefly luciferase. The genetically modified cells were termed JF Luc and TC-71 Luc, respectively. As control cells, we used: (i) A JF Luc derived cell line. This JF based cell line which was termed JF Luc^GD2 low^ was selected as it had downregulated GD2 expression during cell culture (after around 320 passages). The downregulation was confirmed by FACS analysis (Supplementary Figure 1A). (ii) A previously established PC3 Luc cell line which was tested negative for GD2 expression (Supplementary Figure 1B). As shown in Figure 6A, 6B) all the three α-GD2 TMs were able to redirect UniCAR T cells to both GD2 positive cell lines (Figure 6A, 6B), UniCAR CD28/ζ), leading to an efficient lysis. The killing of the target cells via UniCAR T cells was strictly dependent on the presence of the respective TM (Figure 6A, 6B, α-GD2 TM (VL-VH), α-GD2 TM (VL-VH-Li), and α-GD2 TM (VH-VL-Li)). No killing of the target cells was observed with UniCAR T cells in the absence of a TM Figure 6A, 6B), w/o TM). T cells modified with the UniCAR stop vector Figure 6A, 6B), UniCAR Stop) or the EGFP encoding vector control Figure 6A, 6B, Vector control) also did not lyse the target cells. Killing of the target cells was dependent on the presence of GD2 on the surface of target cells (Figure 6A, 6B). There was almost no lysis of the GD2 low expressing JF cells (Figure 6C, JF Luc^GD2 low^) and PC3 Luc cells (Figure 6D, PC3 Luc). Consequently, all three α-GD2 TMs were able to redirect UniCAR T cells in a target-dependent and –specific manner.

### Estimation of range of working concentrations for UniCAR T cells armed with the anti-GD2 TMs

**Figure 7:**
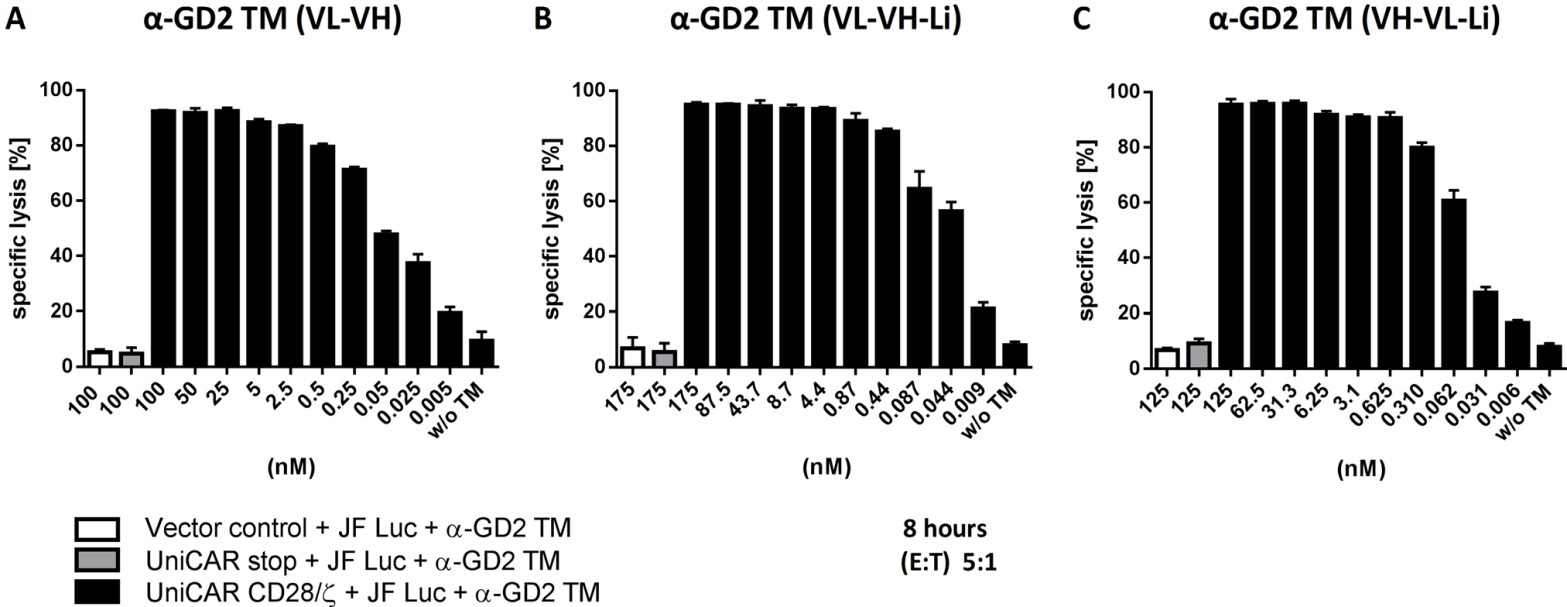
Estimation of range of working concentrations for the α-GD2 TMs Human T cells were transduced with UniCARs containing a dual CD28/CD3ζ signaling domain (UniCAR CD28/ζ) or lacking any signaling domain (UniCAR stop) or expressing only EGFP marker protein (Vector control). UniCAR T cells or controls (stop, Vector control) were incubated with GD2 expressing JF Luc cells at an effector to target cell (E:T) ratio of 5:1. The respective TM (**A**) α-GD2 TM VL-VH; (**B**) α-GD2 TM VL-VH-Li; (**C**) α-GD2 TM VH-VL-Li) was added at the indicated concentrations. After 8h of cultivation, target cell lysis was measured and calculated as described under MATERIALS AND METHODS. The presented curves are based on three donors (A) and one additional donor, respectively (B, C).

For the treatment of experimental mice and for clinical use, the TM concentration will vary in dependence on the elimination of the TM. Therefore, we investigated the concentration range in which UniCAR T cells armed with α-GD2 TMs can mediate the lysis of tumor cells. For that purpose, lysis of JF Luc cells by UniCAR T cells was analyzed by titration experiments (Figure 7). UniCAR T cells were incubated with JF Luc cells at an effector to target cell (E:T) ratio of 5 to 1. The respective TM (Figure 7A, α-GD2 TM (VL-VH), Figure 7B, α-GD2 TM (VL-VH-Li), Figure 7C, α-GD2 TM (VH-VL-Li)) was added at the indicated concentration. After 8h of cultivation, target cell lysis was quantified. Based on these titration experiments we also calculated the respective EC50 values: We estimated an EC50 value of 0.06 nM for the TM α-GD2 TM (VL-VH), 0.02 nM for the TM α-GD2 TM (VL-VH-Li), and 0.06 nM for the TM α-GD2 TM (VH-VL-Li).

### Release of cytokines by retargeted UniCAR T cells occurs in a TM- and target-specific manner

**Figure 8:**
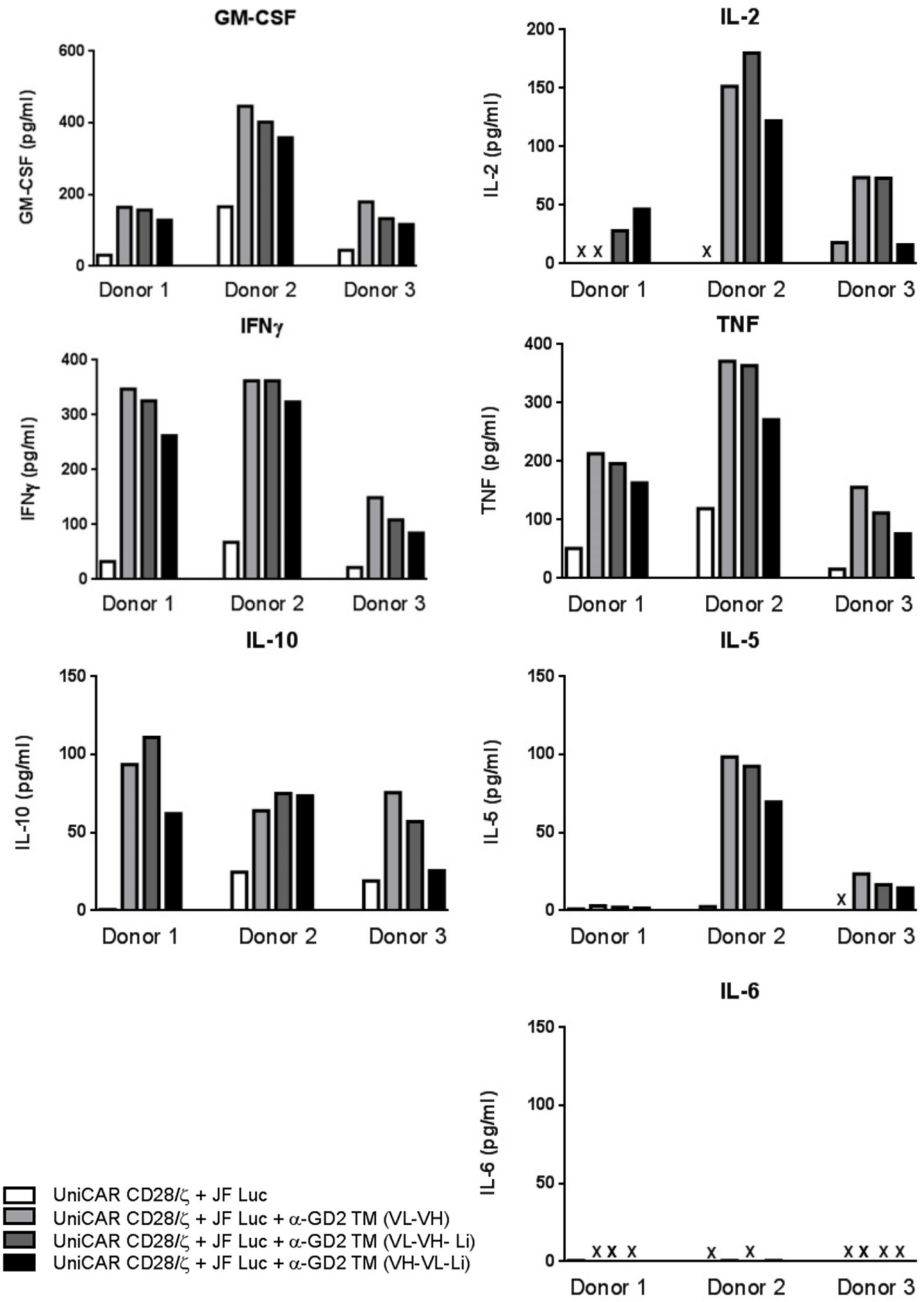
Multiplex analysis of cytokine release from UniCAR T cells armed with 50 nM of the respective α-GD2 TM For analysis of cytokine release, UniCAR CD28/ζ T cells were incubated in the presence or absence of one of the three α-GD2 TMs. Released cytokines were estimated using the MACSPlex Cytokine 12 Kit (see MATERIALS AND METHODS). This bead-based multiplex assay allowed us to detect and quantify the cytokines GM-CSF, IFN-a, IFN-g, IL-2, IL-4, IL-5, IL-6, IL-9, IL-10, IL-12, IL-17A and TNF-a in parallel. Data for the selected cytokines GM-CSF, IFN-g, IL-2, IL-5, IL-10, IL6 and TNF-a are shown. None of the other cytokines was detected.

For analysis of cytokine release, UniCAR CD28/ζ T cells were incubated in the presence or absence of the three α-GD2 TMs. Released cytokines were estimated using the MACSPlex Cytokine 12 Kit. This bead-based assay allowed us to detect and quantify the cytokines GM-CSF, IFN-α,IFN-γ,IL-2, IL-4, IL-5, IL-6, IL-9, IL-10, IL-12, IL-17A and TNF-α in parallel. With the exceptions of GM-CSF, IFN-γ, IL-2, IL-5, IL-10 and TNF-α (Figure 8) no other cytokines could be detected at a significant concentration including IL-6. In summary the analysis of cytokines show: Cytokines were only detected in samples from UniCAR expressing T cells in the presence of (i) target cells and (ii) one of the TMs.

### The UniCAR system triggers anti-tumor effects in experimental mice in dependence on the presence of TMs

**Figure 9:**
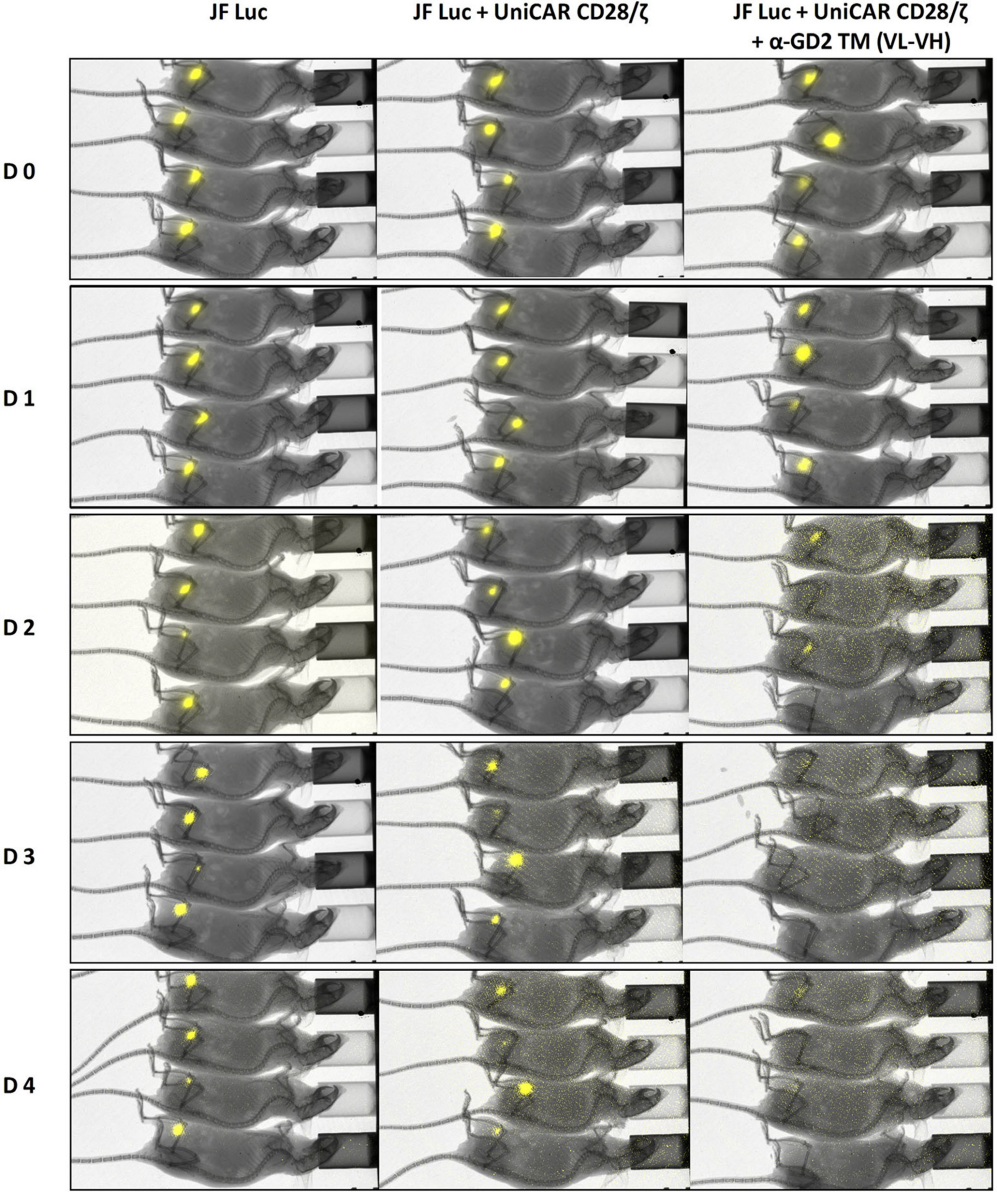
Retargeting of GD2 positive tumor cells in experimental mice JF cells were transduced with firefly luciferase (see MATERIALS AND METHODS) resulting in JF Luc cells. 1 × 10^6^ JF Luc cells were mixed with 1 × 10^6^ UniCAR CD28/ζ T cells and 10 μg per mouse of the α-GD2 TM VL-VH. As “untreated” controls served either 1 × 10^6^ JF Luc cells alone (JF Luc) or JF Luc cells mixed with 1 × 10^6^ UniCAR CD28/ζ T cells without the TM (JF Luc+UniCAR CD28/ζ). The respective mixture (100 µl) was injected subcutaneously into female NMRI-Foxn1nu/Foxn1nu mice resulting in three groups of animals each consisting of four mice. Luminescence imaging of anesthetized mice was performed 10 min after i.p. injection of 200 µl of luciferin (15 mg/ml) starting at day zero (D0), followed at day one (**D1**), day two (**D2**), day three (**D3**), and day four (**D4**).

Comparing the data obtained so far for the three different α-GD2 TMs, it is obvious that all three TMs have similar properties and all three worked comparably well for retargeting UniCAR T cells to GD2 positive tumor cells. In order to reduce the number of experimental animals, we therefore decided to limit *in vivo* experiments to a single α-GD2 TM. For the mouse experiment we selected the α-GD2 TM (VL-VH). In order to show TM-dependent *in vivo* anti-tumor activity of UniCAR T cells against GD2-positive tumor cells, we tested anti-tumor activity in our recently established UniCAR mouse model [36, 37]. For this purpose, 1x10^6^ JF Luc cells were mixed with 1x10^6^ UniCAR CD28/ζ T cells and 10 μg per mouse of the α-GD2 TM (VL-VH) (Figure 9, JF Luc+UniCAR CD28/ζ +α-GD2 TM). As “untreated” controls served either 1 × 10^6^ JF Luc cells alone (Figure 9, JF Luc) or mixed with 1 × 10^6^ UniCAR CD28/ζ T cells without any TM (Figure 9, JF Luc+UniCAR CD28/ζ). The respective mixture (100 µl) was injected subcutaneously into female NMRI-Foxn1nu/Foxn1nu mice resulting in three groups of animals each consisting of four mice (Figure 9). Each group of mice was analyzed in parallel for luciferase activity starting at day zero (Figure 9, D0), followed at day one (Figure 9, D1), day two (Figure 9, D2), day three (Figure 9, D3), and day four (Figure 9, D4). As shown in Figure 9, already at day three, no significant luciferase activity was detectable in all the treated animals while luciferase activity was easily detectable in control mice. These data indicate that UniCAR T cells armed with the α-GD2 TM can also eliminate GD2-positive tumor cells *in vivo*.

### PET imaging of an anti-GD2 TM in tumor bearing mice

**Figure 10:**
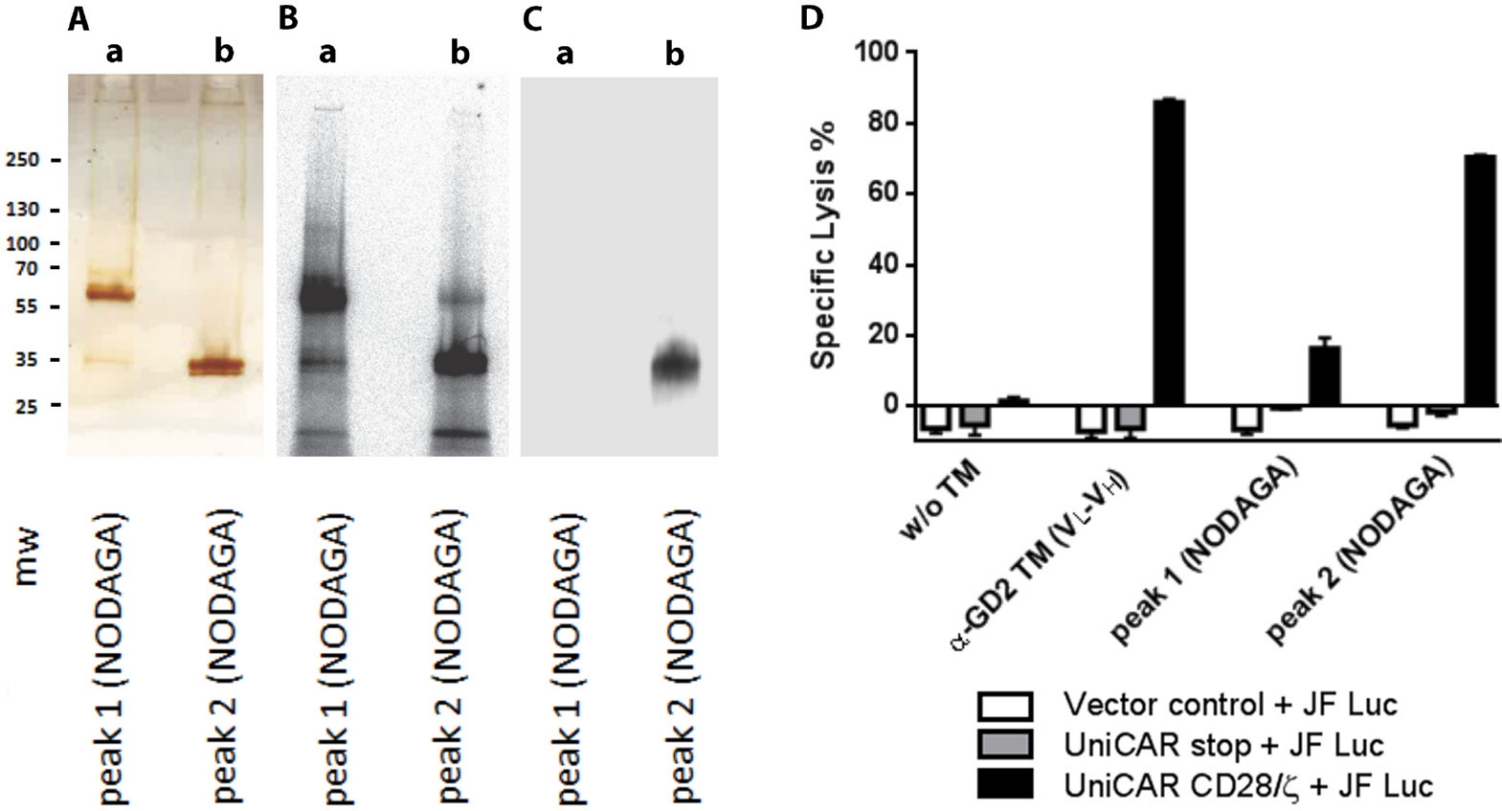
Preparation of protein samples for PET imaging According to Figure 3 the Nickel affinity purified α-GD2 TM is contaminated with HMWs. As these contaminating proteins could interfere with PET imaging, we separated the protein sample by HPLC size exclusion chromatography. Two peaks (termed peak 1 and peak 2) were obtained (see also Supplementary Figure 2). Both protein samples were conjugated with NODAGA and (**A**) analyzed by SDS-PAGE followed by silver staining, (**B**) labeled with ^64^Cu, separated by SDS-PAGE and analyzed by autoradiography, (**C**) analyzed by SDS-PAGE/immunoblotting against the mAb directed to the UniCAR epitope, and (**D**) analyzed for functionality in lysis assays. Lysis of JF Luc cells by T cells modified to express UniCARs (UniCAR CD28/ζ) was estimated in the presence of 50 nM of the respective TM including the Nickel-affinity purified α-GD2 TM (VL-VH) sample, the peak 1 fraction conjugated with NODAGA (Peak 1 NODAGA) or the peak fraction 2 conjugated with NODAGA (Peak 2 NODAGA). As controls, target cells were incubated with either UniCAR T cells in the absence of a TM (w/o TM), or T cells modified with the UniCAR stop vector (UniCAR Stop) or the EGFP encoding vector control (Vector control). Mw, molecular weight marker.

For using of a TM in the context of the UniCAR system the respective TM has to be (i) enriched at the tumor side, and (ii) rapidly eliminated to allow a rapid shut off if unwanted side effects occur. In order to show *in vivo* binding at the tumor side and to estimate the clearance PET analysis was performed. For this purpose the TM had to be further purified as one could expect that otherwise the HMWs contaminating the TM (see also Figure 3) would influence the imaging results. Using size exclusion HPLC chromatography we were able to separate the protein sample into two major fractions (termed peak 1 and peak 2, Supplementary Figure 2). Based on the above described HPLC data (Figure 3) we expected that peak 1 contains the contaminating HMWs while peak 2 should represent the TM. Both protein samples (peak 1 and peak 2) were conjugated with NODAGA (for details see MATERIALS AND METHODS). Prior to PET imaging, the conjugated samples were (i) analyzed by SDS-PAGE followed by silver staining, (ii) labeled with ^64^Cu, separated by SDS-PAGE and analyzed by autoradiography, (iii) analyzed by SDS-PAGE/immunoblotting against the mAb directed to the UniCAR epitope, and (iv) analyzed for functionality in lysis assays. After silver staining both protein samples showed one major protein band (Figure 10A). For the major protein band in peak 1 we calculated a molecular weight of around 68 kDa, for peak 2 a molecular weight of around 33 kDa which is in a good agreement with the theoretical molecular weight of the α-GD2 TM (30 kDa). Peak 1 contained traces of the 33 kDa protein and *vice versa* which is not unexpected as the size exclusion chromatography did not perfectly separate the two peaks and the two peaks were slightly overlapping (see also Supplementary Figure 2). Nonetheless, the remaining contaminations were calculated to be below 1% and may be neglected. Comparable data were obtained when the NODAGA-conjugated samples were complexed with ^64^Cu, separated by SDS-PAGE, and analyzed by autoradiography (Figure 10B). Again the two major bands in the respective protein sample were detected and in addition, the mentioned minor contaminations. After SDS-PAGE/immunoblotting, the mAb against the UniCAR epitope reacted only with the 33 kDa protein present in peak 2 but not with the HMW protein of 68 kDa in peak 1 (Figure 10C) although similar protein amounts of the 33 kDa protein and the 68 kDa protein were present in the respective analyzed sample. Consequently, the 33kDa protein represents the α-GD2 TM while the 68 kDa protein should be unrelated to the α-GD2 TM. This is in agreement with the immunoblotting results already presented in Figure 2B. Moreover, this interpretation is in line with the HPLC data presented above in Figure 3 in which we showed that similar HMWs can be isolated from the unmodified CHO cell line not expressing an antibody reading frame. The mAb against the UniCAR epitope failed to react with the 33 kDa protein contamination which is present in peak 1 as determined by silver staining and autoradiography. So either this 33 kDa protein is unrelated to the α-GD2 TM or alternatively the sensitivity of the immunoblotting procedure was not sufficient to detect these traces of α-GD2 TM.

In order to support the interpretation, that the 33 kDa protein represents the α-GD2 TM and the 68 kDa protein is unrelated to the TM, we estimated the capability of the proteins present in peak 1 and peak 2 to retarget UniCAR T cells to GD2 positive target cells. As expected, the NODAGA conjugated peak fraction 2 (Figure 10D, Peak 2 NODAGA, UniCAR CD28/ζ) contains the highest retargeting activity leading to an efficient lysis of the JF Luc target cells (Figure 10D, Peak 2 NODAGA, UniCAR CD28/ζ). The lysis is close to the original activity of the unmodified α-GD2 TM (VL-VH) (Figure 10D, α-GD2 TM (VL-VH), UniCAR CD28/ζ). There is also some but not significant lysis slightly above the background mediated by the NODAGA conjugated peak 1 fraction (Figure 10D, peak 1 NODAGA, UniCAR CD28/ζ) which could easily be caused by the contamination of the peak 1 with the peak 2 fraction (see also Discussion). No killing of target cells was observed with UniCAR T cells in the absence of a TM (Figure 10D, w/o TM), or by T cells modified with the UniCAR stop vector (Figure 10D, UniCAR Stop) or the EGFP encoding vector control (Figure 10D, Vector control). Consequently, the conjugation of the α-GD2 TM with NODAGA had not destroyed the function of the TM.

Altogether these data support the interpretation that the 33 kDa protein identified in the peak 2 fraction represents the α-GD2 TM and at least the majority of the HMWs in peak 1 are host cell or medium derived contaminations. Moreover significant amounts of dimers or oligomers should also not be present in the peak 1 and therefore in the α-GD2 TM fraction.

**Figure 11:**
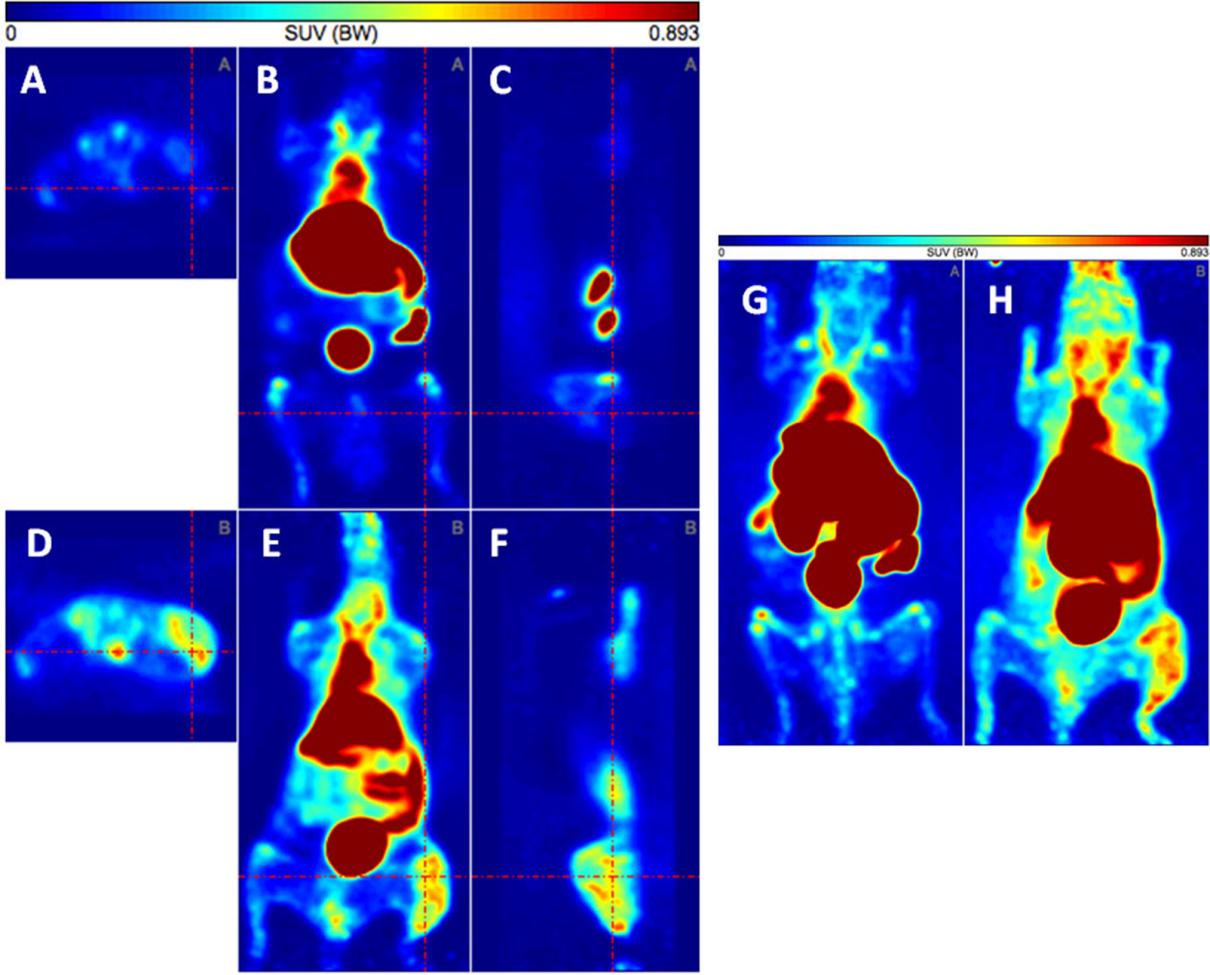
PET analysis of tumor bearing mice to estimate the uptake of the protein fractions of peak 1 (**A–C, G**) and peak 2 (**D–F, H**). Both fractions were conjugated with NODAGA and labeled with ^64^Cu. Uptake was estimated 90 min after i.v. injection (integrated frames from 60 min to 120 min). (A) peak 1, transaxial section, (B) peak 1, coronal section, (C) peak 1, sagittal section, (D) peak 2, transaxial section, (E) peak 2, coronal section, (F) peak 2, sagittal section, (G) peak 1, maximum intensity projection, (H) peak 2, maximum intensity projection. Two of four analyzed mice are shown. The tumor is located where the red lines cross.

**Figure 12:**
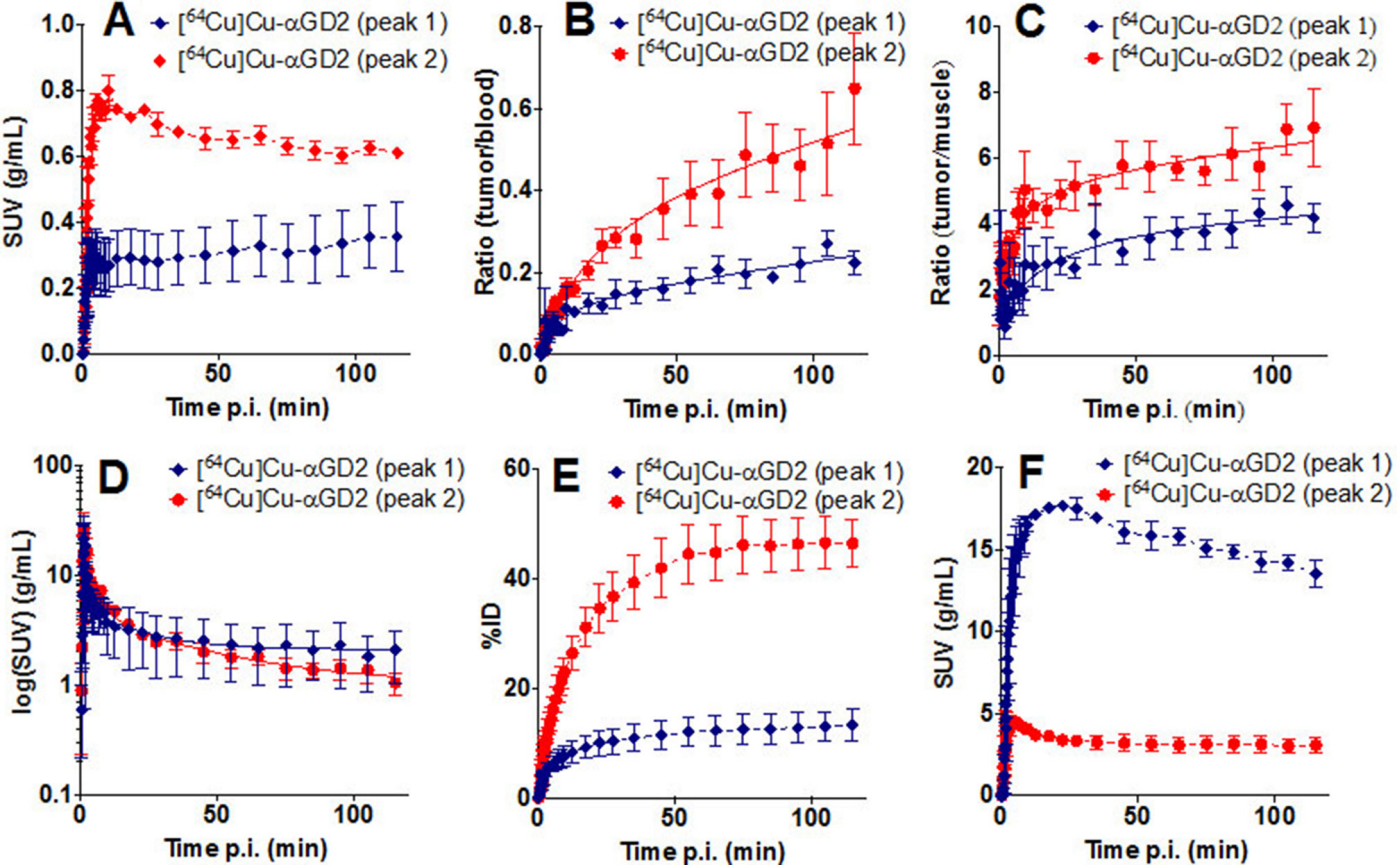
Time activity curves of dynamic PET analysis of tumor bearing mice to estimate the distribution of the protein fractions of peak 1 and peak 2 labeled with ^64^Cu (**A**) activity concentration in the tumor (SUV), (**B**) ratio tumor to blood, (**C**) ratio tumor to muscle, (**D**) blood clearance (log(SUV)), (**E**) renal uptake shown as activity amount as sum kidney + bladder) (%ID), (**F**) liver uptake as activity concentration (SUV).

Finally, small animal PET imaging was performed using both peak fractions conjugated with NODAGA and labeled with ^64^Cu. For this purpose JF Luc tumors were established in NMRI-Foxn1nu/Foxn1nu mice and the uptake of [^64^Cu]Cu-labeled samples derived from either peak 1 (Figure 11A–11C, 11G) or peak 2 (Figure 11D–11F, 11H) was estimated by PET imaging 90 min (integrated frames from 60 to 120 min) after application of the respective sample. PET data show a labeling of the established tumor with the [^64^Cu]Cu-labeled peak 2 fraction (Figure 11D–11F, 11H) representing the α-GD2 TM but not with the peak 1 fraction (Figure 11A–11C, 11G) representing the HMWs. These results are also in agreement with the dynamic PET analysis (Figure 12) for which data points were collected over a time range of 120 min. As shown in Figure 12A the [^64^Cu]Cu-labeled peak 2 fraction is rapidly and efficiently taken up in the tumor in contrast to the [^64^Cu]Cu-labeled peak 1. The enrichment of the peak 2 fraction in the tumor is also supported from the estimated tumor/blood (Figure 12B) and tumor/muscle (Figure 12C) ratios. Both samples are rapidly eliminated as shown in Figure 12D with a half-life of 3.3 min and 27.5 min of the fast and slow distribution and elimination phase for the peak 2. Interestingly, the peak 1 showed an even faster clearance with a half-life of 1 min and 13.3 min of the fast and slow distribution and elimination phase. Most intriguing, however, the elimination pathways of both peak fractions are different (Figure 12E, 12F). As shown in Figure 12E, like other TMs, the peak 2 is mainly eliminated via the kidneys but less extended via the liver (Figure 12F). In contrast, peak 1 is primarily eliminated via the liver (Figure 12F) but not through the kidneys (Figure 12E).

## DISCUSSION

Autologous T cells genetically engineered to express tumor-specific antigen receptors can efficiently recognize and kill malignant cells in an MHC independent manner [13, 14, 18–30]. After first clinical evidence of CAR T-cell activity in neuroblastoma [13, 14, 40], clinical studies using CAR T cells directed against CD19 have demonstrated the impressive therapeutic potential of CAR modified T cells in patients [24–27, 30, 41]. Stable clinical remissions of B lineage malignancies could be achieved even in patients after all standard therapies had failed. After antigen contact, the adoptively transferred T cells can expand to high numbers, producing inflammatory cytokines which can lead to a clinically highly significant cytokine storm. In dependence on the tumor load, tumor cell lysis syndrome may also occur. Anti-tumor activity and side effects are related to *in vivo* proliferation and fitness of CAR T cells and also to tumor load. Since the amount of *in vivo* expansion cannot be predicted by the dose of CAR T cells administered to the patient, determining safe and effective CAR T cell doses is difficult. One approach to reducing the risks associated with CAR T cell therapy is to allow for CAR T cell activity to be switched on and off at will after the re-infusion into the patient. Especially in cases of life-threatening side effects after the adoptive transfer of CAR T cells, the activity and function of the cells has to be stopped as fast as possible. Some strategies have already been put forward how dangerous CAR T cells could be eliminated from a patient: E.g. during genetic manipulation of the T cells further gene(s) can be introduced in addition to the gene encoding the CAR. One way is to co-express a nonfunctional truncated EGFR receptor [42]. As the truncated EGFR receptor still contains the epitope that is recognized by the anti-EGFR ab Cetuximab^®^ this already clinically applied mAb can be used to destroy such CAR T cells. Indeed, following this strategy in a mouse model, CD19 CAR T cells expressing the EGFR receptor could be eliminated and the B cells compartment recovered thereafter [43]. Alternatively, CAR T cells could be modified with a suicide gene. The prototype suicide gene system, which was already established more than 15 years ago for donor lymphocyte is the thymidine kinase from the human herpes simplex virus type 1 (HSV-tk) used in combination with its prodrug, the antiviral drug ganciclovir [44]. In clinical studies, however, T cells expressing HSV-tk were rapidly eliminated due to the high immunogenicity of HSV-tk [45]. Alternative suicide genes could be an inducible caspase9 system [46–48] or a mutated form of human CYP4B1 in combination with the prodrug 4-ipomeanol [49]. The obvious draw backs of all these safety switches are their application will finally lead to the loss of the CAR T cells and the time span for elimination will be quite long. In order to (i) avoid complete elimination of the genetically modified CAR T cells and (ii) realize a rapid shut off of the CAR T cells, in 2014 we presented a switchable modular CAR platform termed UniCAR [31]. As already summarized in the introduction section, UniCAR equipped T cells are not directed to a cell surface target and therefore per se inert. They become active only in the presence of a bispecific TM and after the rapid elimination of the TM they will automatically turn off.

In the meantime, related CAR platforms were described [50, 51]. In 2016 so called sCARs were introduced [50]. Like UniCARs sCARs recognize a peptide epitope. Moreover, sCAR TMs are fusion molecules of the peptide epitope and an antibody domain. However, the ab domain is close to a Y-shaped full size ab. Such TMs should have an extended half-life. Thus, their application may be more convenient for patients but their steering might be more critical. Moreover, in contrast to the human UniCAR peptide epitope, the epitope recognized by sCARs is derived from the nuclear yeast protein GCN4 [50]. Another modular platform is using FITC as epitope [51]. Nonetheless, all these approaches support the feasibility of retargeting of tumor cells with modular CAR T cells that can be armed via target modules. The possibility to repeatedly turn such CAR T cells on and off may facilitate their application for targeting of TAAs which are not limited to tumor cells such as the here selected target GD2 thereby avoiding the risk of live long lasting pain as side effects.

Here we presented three TMs which were developed for retargeting of UniCAR T cells to GD2 positive cells. All three TMs were well expressed and had comparable function with respect to lysis of GD2 positive target cells. Consequently, the UniCAR epitope in these TMs is accessible for UniCAR T cells irrespective of additional flexible linker sequences upstream or downstream of the peptide epitope. Although the affinity of the α-GD2 TM VH-VL-Li was weaker compared to the other α-GD2 TMs its retargeting capability did not dramatically differ supporting the original idea of the UniCAR system, namely, that the affinity between the UniCAR and its target epitope is more important for sufficient and comparable retargeting than the affinity between the TM and the TAA.

All TMs when purified via a single Nickel affinity chromatography step were contaminated with HMWs as analyzed by SDS-PAGE and HPLC. Applying the identical purification procedure to cell culture supernatant of non-transduced CHO cells resulted in the isolation of the same contaminations. The HMWs were not reactive with the mAb directed to the UniCAR epitope. Thus these co-purified proteins are either derived from the CHO cells or the fetal calf serum present in the expression media but not related to an α-GD2 TM. Per se the contaminating proteins isolated from wildtype CHO cell culture supernatant did not interfere with the UniCAR system. Thus, for the preclinical evaluation it was not necessary to further purify the TMs. For PET analysis, however, and in order to estimate the clearance of the TM and the capability to enrich at the tumor site, we had to further purify the TM and to remove the contaminating HMWs. In addition this analysis allowed us to analyze for the presence of dimers or oligomers in our α-GD2 TM preparation. Using size exclusion chromatography we were able to sufficiently separate the HMWs from the actual α-GD2 TM. After separation of the anti-GD2 TM sample into two peaks (peak 1 and peak 2), only the 33 kDa protein present in the peak 2 fraction reacted with the mAb directed to the UniCAR epitope although comparable amounts of the 68 kDa (peak 1) and the 33 kDa (peak 2) protein were present in the respective sample analyzed by immunoblotting. Consequently, the 68 kDa protein present in the peak 1 fraction is unrelated to the anti-GD2 TM. As the separation of peak 1 from peak 2 by the size exclusion chromatography step is incomplete, one would expect, that the HMWs (peak 1) should contain traces of the 33 kDa protein (peak 2) and vice versa. Indeed, when analyzing peak 1 and peak 2 samples with silver staining or the even more sensitive technique of autoradiography we can detect a 68 kDa protein contamination in peak 2 and a 33 kDa protein contamination in peak 1. As the 33 kDa protein present in the peak 1 fraction failed to react with the mAb directed to the UniCAR epitope the 33 kDa protein present in the peak 1 fraction is either unrelated to the TM or the concentration of the TM in this fraction is extremely low and below the detection sensitivity of our immunoblotting system. As both silver staining and autoradiography was performed after SDS-PAGE under reducing conditions, in principle we can not completely rule out that the 33 kDa protein detected in peak 1 is derived from a reduced dimer or higher oligomer of the anti-GD2 TM. However, the lack of immunoreactivity of the 33 kDa protein in peak 1 again strongly argues against the presence of significant amounts of such dimer or oligomers in the α-GD2 TM preparation. In summary, all of our data collected so far including by HPLC size exclusion chromatography, immunoblotting, silver staining after SDS-PAGE, autoradiography, dynamic PET analysis, and even functional lysis assays of the separated protein fractions support the interpretations that (i) the α-GD2 TM is present in the peak 2 fraction, (ii) at least the majority of the HMWs is unrelated to the α-GD2 TM and originate from either the expression cell line or components of the expression media (e.g. the FCS), and (iii) the α-GD2 TM sample should not contain significant amounts of dimers or oligomers.

As killing assay in our study we established a bioluminescence assay instead of previously used chromium release assay which was more reliable for both cell lines and accelerated the readout of the killing assay without loss of specificity. In contrast to chromium release assays, cytokine analysis can be performed directly from such a non-radioactive lysis assay. Our data show that lysis of tumor cells and release of cytokines occur in a strict target-dependent- and -specific cross-linkage of UniCAR T cells with target cells. Thus, using the α-GD2 TMs we were able to target the first non-proteinaceous target with the UniCAR platform. Cytokine release was estimated by MACSPlex assay and confirmed by ELISA (Data not shown). In agreement with previous studies, the most relevant cytokines released by UniCAR T cells were the cytokines GM-CSF, IL-2, TNF and IFN-γ. Release of IL-6 could not be detected. Finally, the TM-dependent killing capability of UniCAR T cells armed with selected α-GD2 TM was also confirmed in a previously described xenografted tumor mouse model for UniCARs [36, 37]. According to dynamic PET analysis, the α-GD2 TM enriches at the tumor site and is rapidly eliminated, thus fulfilling all prerequisites required for a TM useful for combination with the UniCAR system.

In summary, our data show that UniCAR T cells can efficiently be armed with TMs against the disialoganglioside GD2. Retargeting of such armed UniCAR T cells to GD2 expressing cells results in an efficient elimination of GD2-expressing tumor cells both *in vitro* and *in vivo*. The pharmacological properties of the TM are favorable for an application together with the UniCAR platform. Therefore, the combination of the here described α-GD2 TMs with UniCAR T cells represents a promising switchable CAR platform for retargeting of GD2 positive tumor cells overexpressing this surface target.

## MATERIALS AND METHODS

### Cell lines

The neuroblastoma cell line JF was kindly provided by Malcolm K. Brenner, Houston, USA. The Ewing’s sarcoma cell line TC-71 was obtained from DSMZ (Braunschweig, Germany). The prostate cancer cell line PC3 as well as the CHO cell line were purchased from American Type Culture Collection. For the bioluminescence based killing assay and the *in vivo* analysis JF and TC-71 cells were transduced to express the gene encoding firefly luciferase (JF Luc, TC-71 luc). Transduction was performed using a lentiviral packaging system as described previously [39]. PC3 cells expressing firefly luciferase were described previously [36]. All these cell lines were cultured in RPMI 1640 medium completed with 10% FCS, 100 U/ml penicillin and 100 µg/ml streptomycin, 2 mM N-acetyl-L-alanyl-L-glutamine, 1% non-essential amino acids and 1 mM sodium pyruvate (Biochrom). Human Embryonic Kidney cells HEK293T (ATCC CRL-11268) used for production of the lentiviral particles were cultured in DMEM medium supplemented with 10% FCS, 100 U/ml penicillin and 100 μg/ml streptomycin, 1% non-essential amino acids (Biochrom). Cells were maintained at 37°C in a humidified atmosphere of 5% CO_2_.

### Construction and expression of recombinant antibodies

All GD2 TMs were constructed based on the previously described anti-GD2 CAR [13]. The variable heavy and light chain sequences were arranged as schematically shown in Figure 1B. The UniCAR epitope (E5B9) which is based on the nuclear autoantigen La/SS-B [52] was fused to the C-terminus of the scFv either directly or flanked by spacer peptides (N-terminal spacer: AAA; C-terminal spacer: ARGGP). Cloning of the α-GD2 TMs into the lentiviral vector p6NST50 was performed as described previously [e.g. 33]. Stable recombinant TM producing CHO cell lines were established and recombinant proteins were purified from cell culture supernatants via Ni-NTA affinity chromatography followed by analysis of protein concentration and purity through SDS-PAGE and immunoblotting as described [53].

### High-performance liquid chromatography

For size exclusion high-performance liquid chromatography (SE-HPLC) analysis 10 to 15 µl samples containing the respective TM were applied to a size exclusion column (Agilent Bio SEC-3 (3 µm, 150 Å, 7.8 × 300 mm) from Agilent Technologies, Böblingen, Germany). HPLC chromatography was performed using a Chromaster HPLC-System 600 (VWR-Hitachi, Darmstadt, Germany). As running buffer we used 100 mM Na_2_HPO_4_ adjusted to pH 7.0 with HCl. Proteins were detected by UV at 280 nm. The same equipment and settings were used to quantitatively separate the Nickel affinity purified anti-GD2 sample into the peaks 1 and 2 but using as running buffer 150 mM Na_2_HPO_4_ adjusted to pH 7.0 instead (flow rate: 1ml / min).

### Generation of UniCAR vectors

The cloning of the humanized anti-La 5B9 scFv [34, 35], generation of the hinge, transmembrane and signaling domain of the UniCAR was recently described in detail [35]. The scFv was fused at the 5′ end of the CD28 coding sequence with a further peptide epitope of 18 aa (7B6-tag) [39, 54] and an additional G_4_S_1_ linker in between the scFv and CD28. The CAR signaling and stop constructs were subsequently cloned into the lentiviral vector p6NST60 [39]. The orf of the CAR constructs were fused to an EGFP orf separated by a 2pA protease site derived from the *Thosea asigna* virus, which allows the translation of a CAR/EGFP fusion protein from a single mRNA in the modified T cells. After the translation the fusion protein is proteolytically cleaved [55].

### Isolation of peripheral blood mononuclear cells (PBMCs), T cell subpopulations and lentiviral transduction

Isolation of primary human T cells from peripheral blood mononucleated cells (PBMCs) out of buffy coats (supplied by German Red Cross, Dresden, Germany), from fresh blood, or apheresis products of healthy donors and patients was performed as described [38]. The study including the consent form was approved by the local ethics committee of the university hospital of the medical faculty of Carl-Gustav-Carus TU-Dresden (EK27022006). Isolated T cells were cultured in RPMI 1640 complete medium supplemented with 200 U/ml IL-2 (Proleukin^®^ S, Novartis Pharmaceuticals, Horsham, UK), 5 ng/ml IL-7 and 5 ng/ml IL-15 (ImmunoTools, Friesoythe, Germany) at densities of 1–2x10^6^ cells/ml. Production of lentiviral particles and transduction of primary human T cells was carried out as described before [39].

### Cytokine-release assay

For activation experiments, 2.5 × 10^4^ gene modified T cells were seeded in 96-well plates in triplets together with 5 × 10^3^ target cells. TMs were added at a concentration of 50 nM. After 24 h cell free supernatants were harvested and analyzed using the MACSPlex Cytokine 12 Kit (Miltenyi Biotec GmbH), a MACSQuant^®^ Analyzer (Miltenyi Biotec GmbH) and the MACSQuantify^®^ software (Miltenyi Biotec GmbH) according to the manufacturer‘s instructions [36, 37].

### Flow-cytometry analysis

Isolated T cells were stained with fluorochrome-labeled mAbs directed against human CD4/FITC (clone VIT4, MiltenyiBiotec), CD3/Vioblue (MiltenyiBiotec) and CD8/APC (clone BW 135/80, MiltenyiBiotec). For analysis of expression of GD2 on JF cells, the cells were stained using the commercial anti-GD2 mAb (Biolegend, San Diego, USA; Clone 14G2a), detected with Alexa Flour 647-labeled goat anti-mouse IgG (Life technologies, Thermo Fisher Scientific). For detection of CAR surface expression T cells were incubated with the mAb 7B6 and subsequently stained with PE-labeled goat anti-mouse IgG (Beckmann Coulter, Krefeld, Germany). Samples were analyzed using a MACSQuant^®^ Analyzer and MACSQuantify^®^ software (Miltenyi Biotec) [56].

### T cell killing assay

For lysis of tumor cells we used a modified bioluminescence based killing assay (Luciferase assay) [57, 58] instead of standard chromium release assay. For this purpose, tumor cell lines used were transduced with the gene encoding firefly luciferase as described above. Prior to performing lysis assays we controlled the GD2 expression on the target cells by FACS analysis. Tumor cells with a density of 5x10^3^ cell/well were co-cultivated with modified T cells (25x10^3^ cell/well) in the presence or absence of the respective TM in 96 well white plates (CHIMNEY WELL, Greiner Bio-One GmbH, Germany) with a total volume of 200 µl of complete RPMI medium. Plates were kept at 37°C in a humidified atmosphere of 5% CO_2_ for 8 hrs and centrifuged for 3 min at 360xg. After centrifugation and removal of RPMI medium, 25 µl fresh RPMI medium was added. Subsequently, 25 µl of ONE-Glo™ Luciferase reagent (Promega, Madison, US) was added to each well and incubated for three min. Bioluminescence was estimated measured for each sample for 1 second with a luminometer (Tecan, Infinite M200 pro, Switzerland) as relative light units (RLU) (sample RLU). For estimation of maximal killing, cells were treated with 5% Triton-X100 (maximal killing RLU). For measuring of spontaneous death, target cells were incubated without T cells or TMs (spontaneous death RLU). Percent specific lysis was calculated using the following equation: % specific lysis = 100x(spontaneous cell death (RLU) – sample (RLU))/(spontaneous death (RLU) – maximal killing (RLU)).

### Antibody conjugation

After purification of the α-GD2 TM by Ni-NTA affinity chromatography, the protein sample was separated by SE-HPLC (see above) resulting in two major protein peaks termed peak 1 and peak 2. For immuno-PET imaging studies (see below) both protein peaks were conjugated with the chelator NODAGA. Prior to the conjugation, the protein samples were concentrated and buffer was exchanged by 0.1 M borate-buffered saline (pH 8.5) using spin filtration (three times at 7°C and 4000 rpm; Amicon Ultra-4, 10,000 MWCO). Then forty equivalents of 2,2′-(7-(1-carboxy-4-((4-isothiocyanatobenzyl)amino)-4-oxobutyl)-1,4,7-triazonane-1,4-diyl)diacetic acid (p-SCN-Bz-NODAGA) ester were added to the respective protein solution (1 mg/mL). The mixture was left at 4°C for 20 h. Excess chelator was removed by spin filtration with PBS.

### Radiolabeling

The production of ^64^Cu was performed at Cyclone(R) 18/9 (Helmholtz-Zentrum Dresden-Rossendorf) in a ^64^Ni(p, n) ^64^Cu nuclear reaction with specific activities of 150–250 GBq/mmol Cu diluted in HCl (10 mM). [59]. For radiolabeling of both protein samples (representing HPLC peak 1 and peak 2 conjugated with NODAGA) with ^64^Cu, the pH of the ^64^Cu solution was adjusted to pH 5.2 using NH_4_OH and 1.6 nmol of the respective protein sample were added. The respective mixtures were shaken at 37°C for 30 min. Then 1 µmol EDTA was added and the respective radiolabeled sample was separated by spin filtration with PBS. The labeling process was monitored using instant thin-layer chromatography (ITLC). After chelating, the reaction mixture was supplemented with EDTA, and the radiolabeling efficiency was determined using both ITLC and SE-HPLC. SDS-PAGE of the labeled conjugates, followed by silver staining and autoradiography was performed to further evaluate the specific conjugation.

### Optical- and immuno-PET imaging of tumor xenograft mouse models

All animal experiments were carried out at the Helmholtz Zentrum Dresden Rossendorf (HZDR) according to the guidelines of German Regulations for Animal Welfare and have been approved by the Landesdirektion Dresden (24–9165.40–4, 24.9168.21–4/2004–1). Four week old female NMRI-Foxn1nu/Foxn1nu mice were purchased from JANVIER LABS (St. Berthevin, France). General anesthesia was induced with 10% (v/v) and maintained with inhalation of 8% (v/v) desflurane (Suprane, Baxter, Germany) in 30/10% (v/v) oxygen/air. Luminescence imaging (exposure times 1 s, 10 s, and 60 s) was performed using a dedicated small animal multimodal imaging system (Xtreme, Bruker, Germany) 10 min after i.p. injection of 200 µl of luciferin (15 mg/ml) (Thermofisher, Dreieich, Germany). In parallel an X-RAY photograph was taken from the same animals at the same position.

For immuno-PET imaging and biodistribution analysis, naive, athymic male nude (NMRI-Foxn1nu /Foxn1nu) mice (Janvier, France), aged 5–8 wk, were inoculated subcutaneously in the right hind flank with 1x10^6^ JF-Luc cells in PBS. Six to eight weeks after cell inoculation animals bearing tumors between 100 and 500 mm^3^ as measured by a caliper and visual inspection were selected for PET or biodistribution studies.

To evaluate a tumor targeting capability of both [^64^Cu]Cu-(NODAGA)-labeled protein samples, immuno-PET imaging was performed in four JF-Luc NMRI nu/nu tumor bearing mice. Approximately 3.7 MBq of the respective protein sample were intravenously injected into a lateral tail vein of the mice. Dynamic scans were acquired over the following two hrs. Static scans were obtained 90 min after injection using a small animal PET/CT scanner (NanoPET/CT, Mediso). Quantitative data were expressed as SUV, activity concentration normalized to the body weight, that is defined as tissue concentration (MBq/ml)/injected dose (MBq)/the body weight (g) in (g/ml), tumor to muscle and tumor to blood ratios. Images were visualized using ROVER software (ABX GmbH). The time-activity concentration curves were calculated as average ± SEM.

### Statistical analysis

Statistical analysis was performed with GrapPad Prism software version 6.0 (GraphPad Software Inc., La Jolla, CA, USA).

## SUPPLEMENTARY MATERIALS FIGURES


