# Supplementary material for: Development of novel target modules for retargeting of UniCAR T cells to GD2 positive tumor cells
Source: Oncotarget. 2017 Sep 18;8(65):108584–603. doi: 10.18632/oncotarget.21017 (PMC5752466; doi:10.18632/oncotarget.21017)
Supplement: Supplementary file 1 [file oncotarget-08-108584-s001.pdf]

Development of novel target modules for retargeting of UniCAR T cells to GD2 positive tumor cells

SUPPLEMENTARY MATERIALS

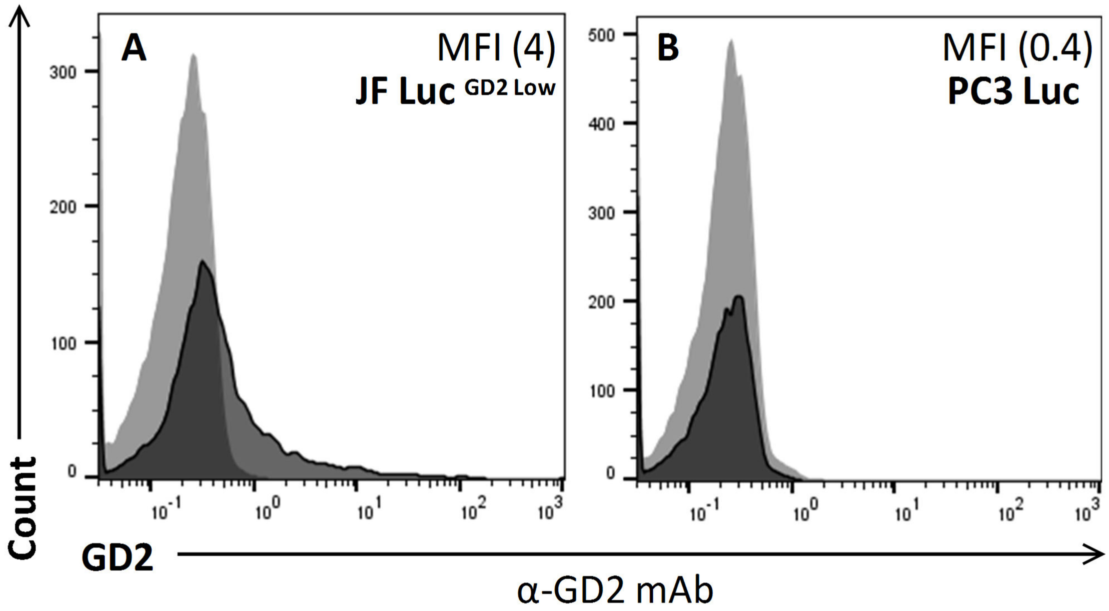

Supplementary Figure 1: Analysis of expression levels of GD2 on control cells. (**A, B**) Expression of GD2 was estimated by FACS analysis: Cells were stained with either a commercially available α-GD2 mAb and detected with Alexa Flour 647-conjugated anti-mouse-IgG mAb (dark graphs) or with the negative control Ab (light graphs). (A) Over time in cell culture JF cells downregulate the expression of GD2. These cells were termed JF LucGD2 lowcells. (B) PC3 cells. (MFI) mean fluorescence intensity of stained cells.

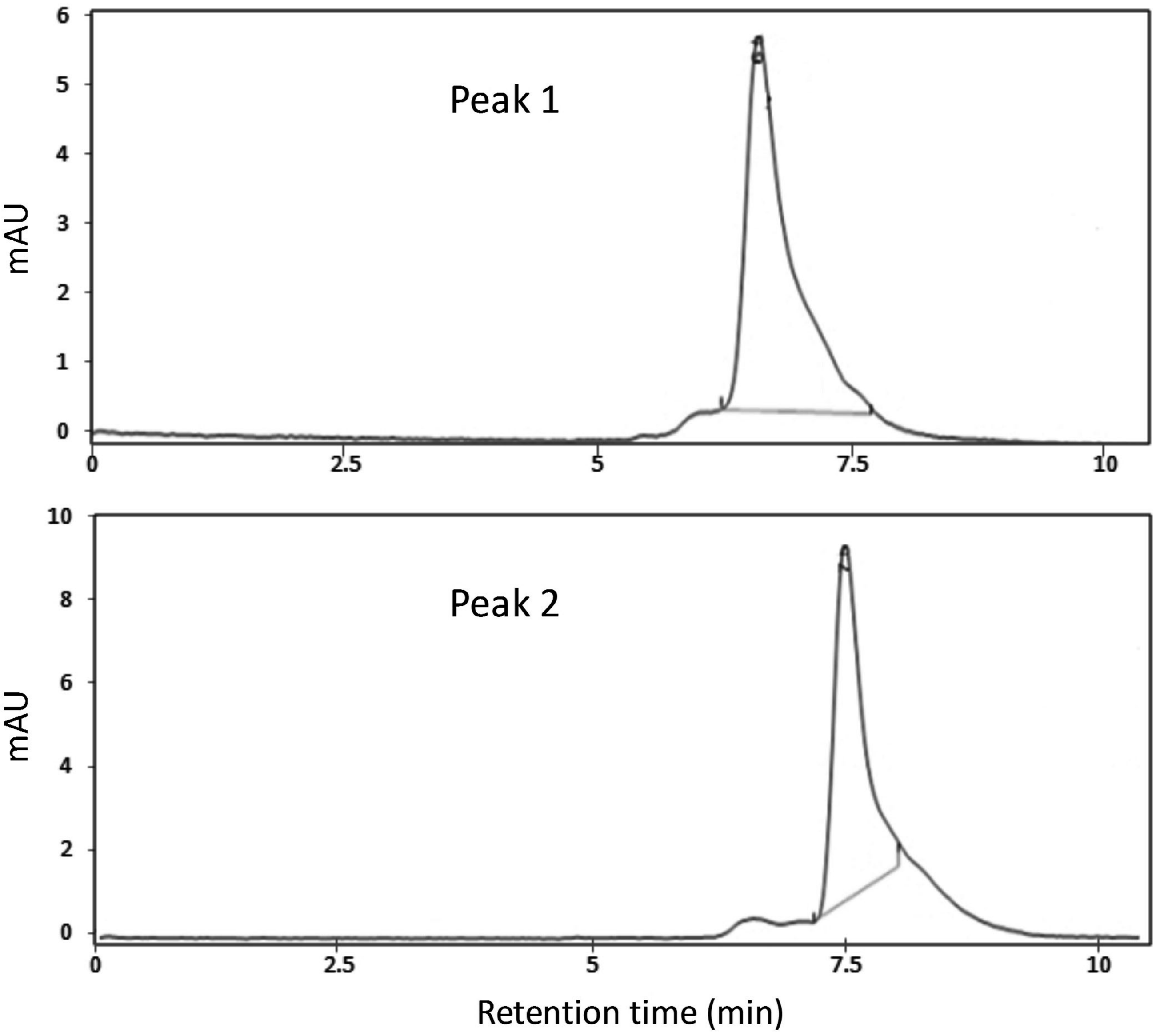

Supplementary Figure 2: HPLC size exclusion chromatography of the purified α-GD2 TM. Nickel affinity purified α-GD2 TM was separated by size exclusion chromatography into two protein fractions termed peak 1 and peak 2. The separated fractions were re-chromatographed by size exclusion HPLC chromatography (see MATERIALS AND METHODS, [37]).
